# Physical Activity Interventions for Mental Health Among Youth in South Africa: A Scoping Review

**DOI:** 10.3390/ijerph23020243

**Published:** 2026-02-14

**Authors:** Osia Livhuwani Munyangane, Lebogang Faith Thaga, Ronald Vele

**Affiliations:** 1 Department of Indigenous Knowledge Systems, Faculty of Humanities, Social Science, University of Venda, Thohoyandou 0950, South Africa; 2 Department of Biokinetics, Recreation and Sport Science, Faculty of Health Sciences, University of Venda, Thohoyandou 0950, South Africa; lebogang.thaga@univen.ac.za; 3 Department of Psychology, Faculty of Health Sciences, University of Venda, Thohoyandou 0950, South Africa; ronald.vele@univen.ac.za

**Keywords:** exercise, mental health, physical activity, youth

## Abstract

Mental health problems are increasing among young people in South Africa due to diverse determinants such as, poverty, social shame, and lack of proper access to health services. Although physical activity is a low-cost and non-medical way to help improve mental health, its effects in rural areas are still not clearly understood. A scoping review was carried out following the PRISMA-ScR guidelines. We systematically searched three online databases (PubMed, CINAHL, and Google Scholar) for studies published from 2014 through 2025. Studies met eligibility criteria if they targeted youth aged 14–35 years living in rural South Africa and reported on physical activity interventions designed for mental health. Two reviewers independently carried out data extraction and came up with the overall result. Overall, 42,384 records were identified, of which only 12 studies met all the specified criteria. The findings of the study were that participation in organised forms of physical activity (including school-based aerobic sessions, community-based walking groups, and charity training programmes) was associated with reductions in mental health issues. Even with these issues, the findings show that physical activity can be a useful, efficient, and practical way to support mental health among rural South African youth. To improve the evidence, strong trials, community-based plans, and sessions at schools and clinics are needed. In addition, policy cooperation across health, education, and sports sectors is essential for lasting impact.

## 1. Introduction

Mental health problems are now one of the major causes of illnesses around the world, and are increasingly affecting teenagers and young adults. Conditions such as depression, anxiety, post-traumatic stress disorder (PTSD), and substance abuse harm a person’s wellbeing and also affect youth schooling, future job chances, and long-term health [1]. The World Health Organisation notes that as many as 20% of young people across the world live with mental health difficulties, yet most do not receive any form of care, especially in low- and middle-income countries (LMICs) where health services are limited and consequently overstretched [2].

In South Africa, the situation is similar. National reports show that about 16.5% of people in the country suffer from mental challenges such as stress, depression, and anxiety during their lifetime, with teenagers and young adults being more affected than older people [3]. More recent studies in some provinces reveal that approximately 15–20% of youth suffer from depression, with about one third of school learners exhibiting signs of anxiety, particularly learners in resource-poor rural settings [4]. These figures indicate a substantial treatment gap, with fewer than one in four individuals who need help actually receiving the appropriate care [5]. These challenges have generated increasing interest in portable low-cost interventions to enhance the mental health of youths outside of traditional clinics.

Regular physical activity has been shown across international studies to reduce signs of depression and anxiety, improve mood, support clear thinking, and help young people build confidence and social relationships [6]. Forms of physical activities such as sport, dance, aerobic exercise, and ordinary recreational games not only support emotional health but also encourage resilience and positive behaviour among youth [7,8,9].

Earlier reviews that examined how physical activity relates to mental health in young people have several limitations. Many of them looked mainly at wealthier or urban environments and do not provide enough guidance for rural communities or places with limited resources [10,11]. A large number of these reviews also depend mainly on cross-sectional research and did not study the range of intervention types or the cultural suitability of programmes in LMICs, including South Africa [12,13]. There is also little combined evidence on how practical these programmes are, how many communities take part in them, or how well they meet rural policy priorities and local needs.

Most of the existing work comes from high-income and urban settings, where schools, clinics, and community facilities make it easier to implement such programmes [10]. Research from these areas shows that physical activity is essential for young people’s health and emotional development [11]. In contrast, rural youth continue to receive very little attention in both research and policy [12]. One reason for studies done in rural places being few in number is that such areas face many barriers that make research challenging [13]. Structural and cultural problems add to this gap, including the lack of safe recreational spaces, limited trained leaders, and weak local infrastructure. Poverty and gender expectations also restrict young people’s chances of taking part in physical activity [14]. In addition, the strong stigma around mental illness together with poor access to formal health care leads to long delays before young people seek help, leaving many without correct diagnoses or treatment [15]. Recent studies warn that the treatment gap for mental health among rural South African adolescents is above 70%, which is a serious public health concern [16].

Studies completed before 2014 have contributed significantly to an understanding of the connection between physical activity and mental health; however, South Africa’s social situation and policy landscape have changed over the last ten years, making it imperative to rely on more recent evidence. Selecting this time period ensures that the findings speak to the present realities of young people, the current health system, and the latest community-based efforts to support local development.

Addressing these inequalities depends on evidence that honestly reflects rural conditions, as programmes developed in urban or clinical settings may not suit rural areas where resources are scarce and where cultural norms strongly shape physical activity and local beliefs about mental illness [17]. Therefore, it is necessary to build an evidence base that fits the current local context so that communities can create relevant, sustainable, and community-led programmes that respond to rural needs and support movement toward universal health coverage (UHC) by improving young people’s access to quality mental health services [18,19,20]. By closely reviewing studies on physical activity programmes for youth mental health in rural South Africa, this scoping review addresses these gaps. Unlike older reviews that were limited or focused mainly on urban areas, this study gathers current intervention strategies, highlights culturally relevant models, and identifies the main weaknesses in the existing evidence. This combined approach connects the aim of this review to the shortcomings of earlier work and offers practical guidance for researchers, service providers, and policymakers to design culturally suitable and scalable interventions that can help to reduce the gap between rural and urban youth mental health outcomes.

### Research Objective

The aim of this scoping review is to identify and outline physical activity programmes designed to improve mental health outcomes for young people in South Africa.

## 2. Materials and Methods

### 2.1. Study Design

This study applies a scoping review method, and is directed by the Preferred Reporting Items for Systematic Reviews and Meta-Analyses extension for Scoping Reviews (PRISMA-ScR) guidelines [21]. The completed PRISMA-ScR checklist used to guide the conduct and reporting of this review is provided as Supplementary Table S1. The process follows the framework first set out by Arksey and O’Malley [22] and later strengthened by Levac et al. [23]. This method was used to arrange the available studies in an organised manner, identify key ideas, list the various available intervention types, identify the gaps in the evidence, and document current research trends linked to physical activity and mental health among rural youth in South Africa.

### 2.2. Protocol and Registration

The protocol for this scoping review was registered beforehand on the Open Science Framework (OSF) to promote transparency and sound methodological practice [24]. This protocol sets out the purpose of the review, the study selection criteria, and the planned steps of the process, ensuring that the review follows accepted standards for scoping reviews, as recommended by the PRISMA-ScR guidelines.

### 2.3. Eligibility Criteria

This review applied the Population–Concept–Context (PCC) framework to guide the selection of suitable studies and included publications from 2014 to 2025. Evidence on the mental health advantages of physical activity has been available for many years; however, this review focuses on the last decade in order to reflect the current South African situation. Over this period, there have been critical shifts in youth mental health policies, school-based health promotion, and community-led physical activity programmes. Limiting the timeframe to this period made it possible to capture contemporary intervention methods, recent trends in youth mental health, and the changing social and cultural influences that shape young people’s participation in physical activity.

Population: The review included studies that focus on young people aged 14 to 35 and living in rural areas of South Africa. This age range aligns with national definitions of “youth” as a stage of life when mental health challenges commonly begin to emerge.
Concept: Relevant studies focused on physical activity or exercise-based programmes designed to improve mental health. These may involve formal or informal activities, including aerobic exercise, sports, recreational play, dance, or community movement programmes.
Context: The review focused on interventions within South Africa that aim to promote mental wellbeing, prevent mental health problems, or support treatments.
Study Design: To ensure broad and comprehensive coverage of the topic, the review considered a variety of study designs, including quantitative, qualitative, and mixed-methods, as well as evidence summaries such as systematic reviews, scoping reviews, and conceptual papers when these were deemed to contribute significant theoretical or contextual insights into physical activity and its relation to mental health among young people in South Africa.

### 2.4. Search Strategy

A comprehensive and systematic search was conducted across multiple electronic databases to identify relevant studies examining physical activity interventions for mental health among rural youth in South Africa. The databases searched included PubMed, CINAHL, and Google Scholar. The search covered the period January 2014 to September 2025 in order to capture the most recent and relevant evidence.

The population guided the search strategy using the Population–Concept–Context (PCC) framework; the population was youth in rural South Africa, the concept was physical activity or exercise interventions, and the context was mental health and wellbeing. Search terms combined key concepts related to physical activity, mental health, youth populations, and rural settings in South Africa.

The search string was as follows:

(“physical activity” OR exercise OR “sport-based intervention” OR “recreational activity”) AND (“mental health” OR depression OR anxiety OR stress OR “psychological well-being”) AND (youth OR adolescent OR “young people” OR “young adult” OR teenager) AND (“rural South Africa” OR “rural communities”).

In addition to peer-reviewed journal articles, selections from the grey literature were included when they were found to be relevant and methodologically sound. These sources were identified through a hand search of reference lists.

### 2.5. Study Selection Process

Two researchers, OLM and LT, independently screened the titles and abstracts of all identified studies using Rayyan version 1.0. They followed the predefined criteria to decide which studies to include or exclude. When OLM and LT disagreed on a study, a third researcher, RV, was consulted to help reach a consensus and ensure that the process was fair. This approach aligns with best practices for reviews, thereby helping to reduce bias and maintain consistency in decision-making.

Cohen’s kappa was not formally calculated to measure reviewer agreement; however, the screening process proceeded smoothly with a high level of consensus, as any minor disagreements were resolved through guided discussions with the third reviewer. Having multiple reviewers and making joint decisions ensured that the selection process was thorough, transparent, and reliable.

Full-text articles were obtained for those studies that had passed the initial screening. These articles were then examined in detail using the same inclusion and exclusion criteria (shown in Table 1). The final set of included studies included those conducted through qualitative, quantitative, and mixed-methods approaches, as well as review articles; this diversity of study types allowed our review to capture a comprehensive range of evidence on physical activity programmes and mental health among young people in South Africa.

### 2.6. Data Extraction

Data from the selected studies were systematically collected using a standardised data extraction form developed beforehand in order to ensure consistency and completeness [25]. The extracted information included:

- Author(s) and year of publication: To track trends in publication and provide contexts for the findings.
- Study design and methodology: To understand the research approaches and assess study quality.
- Sample characteristics: Focus was placed on aspects of age, gender distribution, and geographical location in order to obtain a detailed description of the population.
- Setting: Information about where the intervention took place, such as schools, community centres, or clinics, further establishing the context.
- Type and description of physical activity intervention: Details covered, intervention format, duration, frequency, and method of delivery.
- Types of mental health challenges: These included depression, anxiety, stress, or other forms of psychological ill-health.
- Main findings and conclusions: These discussions summarised the effectiveness of the interventions and highlighted other key insights.
- Data extraction was carried out independently by two reviewers. Any differences were resolved through discussion, or, if needed by consulting the third reviewer, in accordance with best practices for scoping reviews [23].

### 2.7. Quality Assessment

Scoping reviews do not require formal quality assessment; however, the methodological quality of the included studies was assured using the Mixed Methods Appraisal Tool [26] to help judge the reliability of the evidence and guide interpretation of the results. Each study was assessed according to its methodological stringency, with attention given to how clearly the research questions were stated, how well the study design matched the aims, whether the measurement tools were suitable, how sampling was carried out, the completeness of the data, and whether the findings and conclusions were consistent. This organised method allowed for a uniform assessment across the different study types and made it possible to identify both methodological strengths and weaknesses in the evidence base.

Overall, the methodological quality of the studies varied. Some quantitative studies clearly presented their research questions and used suitable outcome measures to assess physical activity or mental health. The qualitative studies often provided valuable contextual understanding of young people’s experiences and views of physical activity programmes, although a few did not fully explain their analytic procedures. Mixed-methods studies were few, although in those that were included, the connection between the qualitative and quantitative parts was often weak or not clearly explained.

### 2.8. Data Synthesis

A narrative synthesis approach was used to summarise and interpret the findings from the selected studies. Extracted data were organised around the core requirements of the review components: population characteristics such as age, gender, and whether rural or urban setting, the types of physical activity interventions implemented, an assessment of the mental health outcomes from these intervention initiatives, and the contextual factors influencing programme delivery and participation.

Given the heterogeneity in study designs, participant groups, intervention types, and outcome measures, numerical pooling of data or meta-analysis was not appropriate, and consequently was not undertaken. Instead, the quantitative and qualitative findings were compared thematically to identify patterns, consistencies, and gaps across studies. This approach is consistent with scoping review methodology and allowed for a broad mapping of the evidence base on the physical activity interventions for youth mental health in South Africa.

### 2.9. Ethical Considerations

Because this review synthesised publicly available evidence and did not involve direct interaction with human participants, ethical approval was not required. However, ethical practices reported in the included studies such as informed consent, protection of minors, confidentiality, and community engagement were noted during the methodological appraisal to ensure that the underlying evidence adhered to recognised ethical standards.

## 3. Results

### 3.1. Study Selection

**Figure 1:**
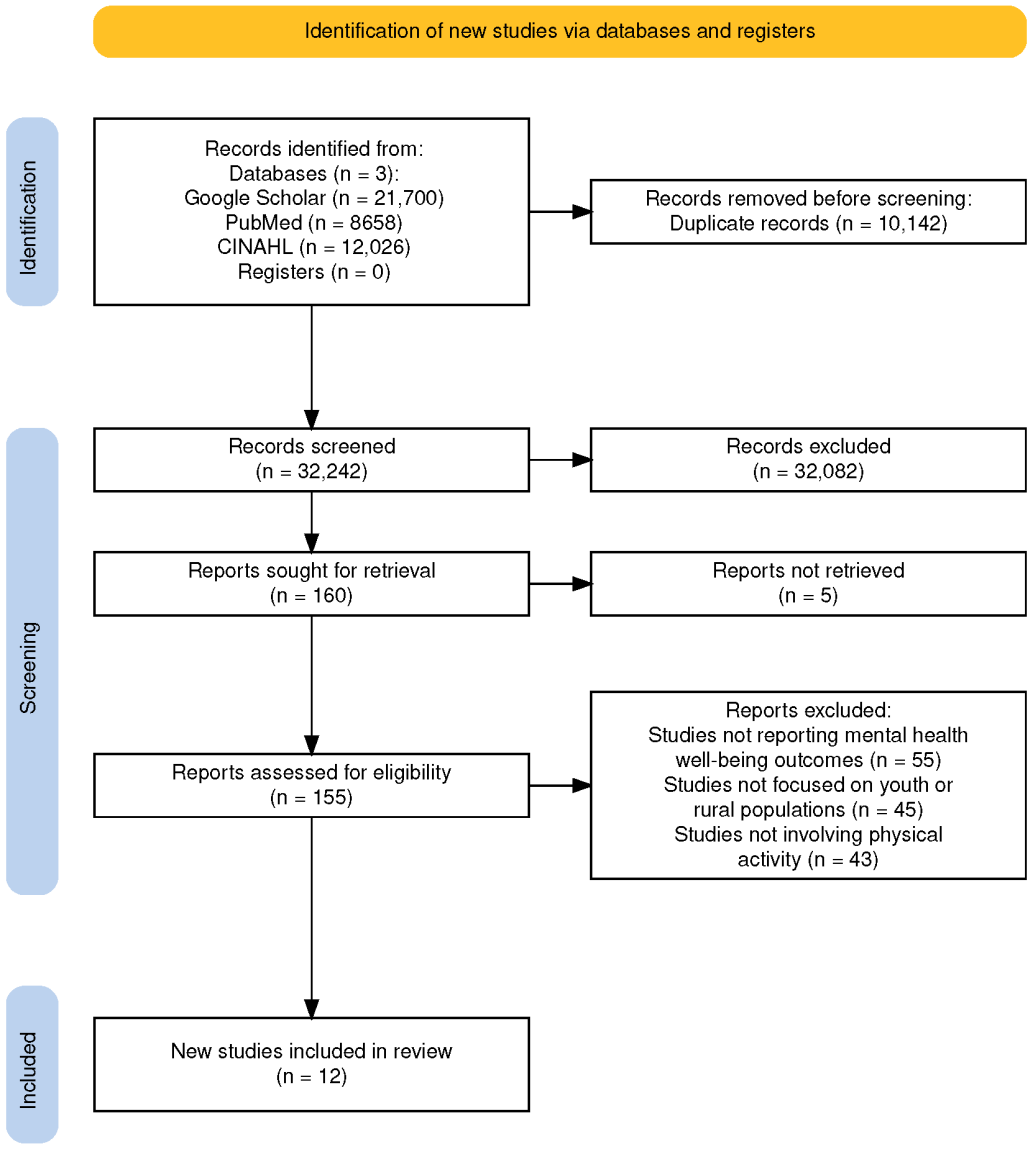
PRISMA-ScR diagram.

A systematic search was conducted across Google Scholar, PubMed, and CINAHL. The search identified 42,384 records (Google Scholar: 21,700; PubMed: 8658; CINAHL: 12,026). After removing 10,142 duplicates, 32,242 records remained for title and abstract screening. Following this stage, 160 articles were selected for full-text review based on relevance to the eligibility criteria. Of these, 12 studies met the inclusion criteria and were included in the final synthesis. A PRISMA-ScR flow diagram summarising the study selection process is presented in Figure 1.

### 3.2. Characteristics of the Selected Studies

The 12 studies selected for this review differed in their design, target groups, settings, and outcomes, showing a range of approaches exploited in exploring the link between physical activity and youth mental health in South Africa. The research designs consisted of five cross-sectional studies [14,16,27,28,29], two qualitative studies [30,31], one intervention development study [32], two systematic reviews [33,34], one scoping review [35], and one conceptual paper [36]. Sample sizes varied, with qualitative studies involving fewer than 50 participants, while the larger cross-sectional quantitative studies involved more than 380 participants. Most studies took place in schools or community settings, while a smaller number focused on healthcare providers or used national datasets. Together, these studies offer an understanding of both personal and structural factors that influence physical activity and mental health among adolescents and young adults.

Populations targeted in these studies were primarily adolescents and youth, with several studies focusing on young women and healthcare professionals [14,16,27,28,29,30]. Studies were conducted across diverse contexts, including urban, peri-urban, and rural, in provinces, such as KwaZulu-Natal, Mpumalanga, and the Eastern Cape. Sampling techniques reflected the study design, ranging from purposive and convenience sampling in qualitative works to survey-based and stratified approaches in quantitative studies [14,30,31].

Cross-sectional studies consistently reported low levels of physical activity, particularly among adolescent girls and youth in rural areas, with sedentary behaviour negatively associated with mental health outcomes. Asare et al. [16] found that higher physical activity levels among adolescents in KwaZulu-Natal were associated with fewer behavioural problems and enhanced pro-social behaviours, highlighting the role of physical activity in resilience-building. Micklesfield et al. [14] reported similarly low engagement in moderate-to-vigorous physical activity among rural adolescents in Mpumalanga, noting socio-economic factors as key determinants of participation. Siduli et al. [29] observed positive associations between physical activity participation, body composition, and mental wellbeing in adolescents from the Eastern Cape, reinforcing the reported diverse health benefits from active lifestyles. Draper et al. [27] reported that high sedentary behaviour among young women in urban Soweto was correlated with poorer mental health outcomes, suggesting that these patterns are likely magnified in rural youth with limited opportunities for physical activity.

The qualitative studies provided in-depth insights into barriers and facilitators of participation. Kinsman et al. [30] used interviews and focus group discussions with rural adolescent girls in Mpumalanga, identifying challenges such as body image concerns and safety along with proposed strategies for increasing engagement. Marais [31] interviewed mental health professionals and highlighted positive attitudes toward integrating physical activity into care, but noted insufficient training and institutional support as factors limiting this integration. Intervention-focused work demonstrated the effectiveness of culturally-tailored programs. Zimu et al. [32] developed the Nyakaza–Move-for-Health program, a school- and community-based intervention that successfully enhanced adolescent engagement, drawing attention to the need for these activities to be culturally sensitive.

Evidence synthesis and conceptual studies underscored the need for structural and policy-level approaches. Vancampfort et al. [34] highlighted the limited availability of policy- and community-level interventions in Sub-Saharan Africa while noting their crucial role in supporting mental health outcomes. Burger et al. [36] proposed integrating sport and exercise psychiatry into South Africa’s mental health systems, advocating for professional capacity-building and efforts to ensure programmes’ feasibility. In a systematic review of global adolescent physical activity interventions, Bermejo-Cantarero et al. [33] confirmed significant improvements in emotional wellbeing and quality of life resulting from structured programs. Kunene and Taukobong [28] examined physical activity among healthcare professionals in rural KwaZulu-Natal and found very low engagement, raising concerns about their role as promoters of active lifestyles. Mumbauer et al. [35] conducted a scoping review of youth-focused interventions in South Africa and recommended culturally relevant and school-based approaches to integrate physical activity into broader mental health promotion strategies.

Across these studies, several consistent themes emerged. Physical activities were positively associated with mental health outcomes, yet engagement with them remains low, particularly among girls, rural youth, and healthcare professionals. Barriers such as safety concerns, body image, cultural norms, and institutional constraints persist, while culturally and contextually tailored interventions show promise in improving participation and outcomes. Methodological limitations, underrepresentation of rural populations, and the predominance of cross-sectional designs highlight significant gaps in the evidence base. Taken together, these studies emphasized the need for scalable, context-sensitive, and inclusive interventions that can effectively support youth mental health. The characteristics of all the selected studies, focusing on design, population, setting, and key findings, are summarised in Table 2.

## 4. Discussion

This scoping review examined evidence on physical activity (PA) interventions aimed at improving mental health among youth in South Africa, with particular attention to rural contexts where mental health needs are high and services remain constrained. The findings confirm the potential of PA as a non-pharmacological strategy for mitigating symptoms of depression, anxiety, emotional distress, and behavioural challenges. However, the review also revealed substantial disparities in access, participation, and intervention effectiveness, highlighting the need for contextually-grounded, culturally-responsive, and system-supported approaches to PA promotion in rural youth populations.

### 4.1. Summary of Key Findings

Across the 12 selected studies, PA consistently demonstrated positive mental health associations such as improved emotional wellbeing, reduced behavioural difficulties, and enhanced prosocial functioning. However, these benefits were not evenly distributed across populations. Rural youth were consistently less active than their urban counterparts, seemingly driven by limited facilities, safety concerns, and restrictive socio-cultural norms, particularly for girls. The quantitative studies revealed clear links between PA levels and mental health outcomes [14,16,29], while the qualitative studies highlighted contextual constraints affecting participation [30,31]. Intervention-focused and conceptual papers further demonstrated that culturally adapted programmes show limited promise, especially in rural settings [32,36]. Collectively, these findings provide a strong evidence base for the role of PA in youth mental health while also exposing significant implementation challenges in South Africa.

### 4.2. Physical Activity and Mental Health Outcomes 

Across the included evidence base, physical activity levels showed a consistent and positive relationship with mental health outcomes among South African youth. Higher participation in moderate-to-vigorous physical activity was associated with fewer behavioural problems and improved psychosocial resilience among adolescents [16]. Similarly, Siduli et al. [29] reported that adolescents with higher activity levels had better mental wellbeing scores and healthier body composition. In rural Mpumalanga, Micklesfield et al. [14] found very low levels of activity among adolescents, with sedentary behaviour negatively associated with psychological functioning.

Urban data reinforced these patterns. Draper et al. [27] observed that high sedentary time among young women in Soweto was linked to poorer mental health profiles, a concern likely to be magnified in rural areas where opportunities for PA are even more limited. Qualitative findings offered further insight; Kinsman et al. [30] showed that body image concerns, identity formation, limited recreational spaces, and cultural expectations shaped both activity participation and emotional wellbeing among rural girls. Marais [31] discovered that mental health providers viewed PA as beneficial but lacked the institutional structures, training, and resources to implement these PA-based interventions.

Intervention-related studies offered promising evidence. Zimu et al. [32] demonstrated that a culturally grounded programme called Nyakaza–Move-for-Health increased adolescent engagement with PA and supported positive psychosocial outcomes. Global evidence further concurs with these findings; for instance, Bermejo-Cantarero et al. [33] reported substantial improvements in emotional wellbeing and health-related quality of life in adolescents participating in structured PA interventions. Conceptual and review studies also provided supporting frameworks. Burger et al. [36] called for integrating sport and exercise psychiatry into South Africa’s mental health system, while Mumbauer et al. [35] advocated for culturally relevant and youth-centred models. At a regional level, Vancampfort et al. [34] underscored the limited availability of structured PA interventions in Sub-Saharan Africa despite strong evidence of their effectiveness.

Taken together, these studies demonstrate that PA has measurable mental health benefits, but that outcomes depend heavily on factors such as access to structured programmes, contextual feasibility, and exposure to supportive environments. Rural adolescents remain disproportionately under-active, indicating a need for tailored interventions to remedy mental health challenges.

### 4.3. Interventions and Context-Specific Models

Intervention-focused studies provided key insights into effective approaches for rural and semi-rural youth. The Nyakaza–Move-for-Health programme [32] illustrates how incorporating cultural practices, family participation, and community ownership can promote sustained engagement. Similarly, the rural Mpumalanga model for adolescent girls [30] cited identity, body image concerns and safety issues as central determinants of participation, emphasising the need for gender-sensitive approaches.

In contrast, urban-based studies such as those conducted in Soweto [36] offer valuable epidemiological insights, but are less applicable in rural settings. Infrastructural advantages of urban areas such as trained facilitators, organised school sports, and greater access to facilities underscore the limitations of applying urban models to rural communities. These differences suggest the need for differentiated context-specific strategies in order to avoid widening existing inequities in mental health and physical activity participation.

### 4.4. Health Systems and Professional Perspectives

System-level constraints also emerged as significant barriers to the integration of PA into mental health services; although clinicians acknowledged the therapeutic potential of PA, they lacked institutional support, training, and operational frameworks to incorporate exercise into clinical practice [31]. Conceptual contributions, for instance the sport-and-exercise psychiatry model proposed by Burger et al. [36], stress the need for specialist roles, clinical pathways, and policy guidance to ensure sustainable implementation. Low PA participation among healthcare providers themselves [28] presents an added challenge, as providers who do not model active lifestyles themselves may be less likely to promote PA to youth. Therefore, strengthening providers’ capacity, workplace wellness, and professional development is essential for effective integration.

### 4.5. Global Comparisons and Broader Insights

International evidence strongly supports the mental health benefits of structured PA in young populations, including reductions in depressive symptoms, improvements in emotional wellbeing, and enhanced quality of life [33]. However, translating global evidence to South African rural contexts requires an understanding of local realities. Rural South Africa faces constraints related to socioeconomic conditions, limited facilities, transport barriers, and gendered social norms which may limit the feasibility of standardised interventions. Comparable findings from rural settings in countries such as Australia and New Zealand demonstrate that structural inequities and cultural expectations do significantly shape PA engagement. These parallels reinforce the need for South African interventions that are explicitly designed for rural populations and grounded in local social and cultural contexts.

### 4.6. Gaps in the Literature

This review highlights several critical gaps. First, most included studies were cross-sectional [14,16], limiting the ability to draw causal inferences. First, few randomised controlled trials (RCTs) or quasi-experimental evaluations tested the efficacy of physical activity interventions for rural youth. Without stronger designs, it is difficult to establish long-term effectiveness or determine optimal intervention components.

Second, rural youth remain underrepresented. Even when rural populations were included, findings were often not disaggregated by geographic location, which reduced their applicability. The studies illustrate this urban focus, leaving rural youth, who are among the most vulnerable groups, overlooked [11].

Third, poor reporting remains a problem. Globally, many studies fail to provide adequate details on intervention frequency, duration, and adherence. This limitation reduces replicability and hinders scalability [9].

Finally, certain vulnerable groups, including out-of-school youth, adolescents with disabilities, and LGBTQ+ populations, are rarely represented; this omission perpetuates inequities and restricts understanding of how physical activity may benefit diverse youth subgroups in rural contexts.

### 4.7. Limitations

This scoping review has several limitations that should be considered when interpreting the findings. Most of the selected studies were cross-sectional, limiting causal inference and offering little insight into long-term intervention effects. Rural populations remained underrepresented; in addition, few studies disaggregated their results by location, gender, or other subgroups such as out-of-school youth or vulnerable adolescents with disabilities. Poor reporting of intervention details such as duration, intensity, and adherence further limits replicability. The diversity of study designs prevented meta-analysis and made cross-study comparisons challenging. Methodological limitations of the review itself also apply. Restricting the search to studies published between 2014 and 2025 may have excluded earlier foundational research, although this timeframe was specifically selected in order to reflect recent policy developments and intervention models. While the review offered a comprehensive database and grey literature list, some relevant studies may still have been missed. Screening was performed independently by two reviewers, with a third resolving discrepancies; however, inter-rater reliability was not formally calculated. These factors highlight the need for cautious interpretation and signals the urgency of future research.

### 4.8. Implications for Research, Policy, and Practice

The evidence from this review affirms physical activity as a critical and low-cost strategy for supporting youth mental health in South Africa, particularly in rural areas. In order for programs to be effective, however, they must be community-driven and culturally sensitive; additionally, they must engage youth, families, and local leaders in their design and implementation. Schools provide a natural delivery platform due to their reach and potential for structured and sustainable programming; similarly, culturally tailored school–community partnerships have demonstrated improved outcomes [37]. Policy integration is essential for sustainability, as this approach enables the linking of mental health to education and sports development strategies, thereby allowing for the mobilization of resources and institutional support. Building capacity among mental health professionals is crucial for embedding physical activity within both clinical and community settings [31,36]. Future research should prioritize longitudinal and experimental studies in order to establish causal relationships and evaluate intervention effectiveness, particularly in rural contexts. Mixed methods approaches can clarify how cultural, gender, and infrastructural factors influence participation, while digital solutions such as tele-health services can offer innovative means of overcoming geographic and accessibility barriers. Hence, coordinated efforts across the domains of community engagement, school integration, and policy support are vital to reducing rural–urban disparities while providing accessible and non-stigmatizing pathways to wellbeing.

## 5. Conclusions

This scoping review found consistent evidence that physical activity improves mental health outcomes among the youth in South Africa, reducing depression, anxiety, and behavioural challenges while enhancing resilience and pro-social behaviours. However, participation levels remain low and rigorous intervention trials targeting rural youth are scarce. Policy and practice efforts should focus on integrating physical activity into youth mental health promotion through schools, community centres, and primary healthcare systems, particularly in rural areas. Cross-sector collaborations between health, education, and sports organisations are essential to ensuring sustainability, scalability, and equity. Building the capacity of healthcare professionals to deliver and advocate for PA-based interventions will further strengthen implementation.

Looking forward, these findings testify to the need for integrated health and education interventions that will embed culturally tailored physical activity programs within broader youth development agendas. In LMIC contexts such as South Africa, such strategies can reduce rural–urban disparities, provide non-stigmatising pathways to care, and promote long-term wellbeing for vulnerable youth populations.

This review was conducted in accordance with a prospectively registered protocol on the Open Science Framework.

## Figures and Tables

**Table 1:** Inclusion and exclusion criteria.

Criteria	Inclusion	Exclusion
Timeframe	Studies published between 2014 and 2025	Studies published before 2014
Study Design	All empirical study designs: quantitative, qualitative, or mixed methods	Editorials, commentaries, and non-peer-reviewed literature
Population	Youth (14–35 years) in South Africa	Studies focused only on older people/children’s
Intervention	Any physical activity or exercise intervention	Interventions focused only on elite or professional athletes
Outcomes	Studies that report on mental health outcomes.	Studies that do not assess mental health outcomes
Language	Articles published in English	Articles published in other languages without an English translation

The Timeframe (2014–2025) was chosen in order to reflect contemporary evidence and policy developments affecting youth mental health and physical activity in South Africa.

**Table 2:** Summary of included studies.

Author (Year)	Design	Location	Sample and Sampling Technique	Instrument	Outcomes	Intervention
[16]	Cross-sectional	South Africa–KwaZulu-Natal	187 adolescents; convenience sample	Strengths & Difficulties Questionnaire, PA recall	Behavioural challenges, prosocial behaviour	None (PA levels and mental health correlation)
[30]	Qualitative (model development)	South Africa–rural Mpumalanga	Adolescent girls; purposive sampling	Interviews, FGDs	Barriers to activity, identity, and body image	Model for promoting PA among rural girls
[14]	Cross-sectional	South Africa–Agincourt HDSS, Mpumalanga	381 adolescents; random cluster sampling	Self-reported PA, SES, and BMI	MVPA, sedentary behaviour	None (baseline behaviour analysis)
[34]	Systematic review	Sub-Saharan Africa	Studies across SSA countries	Not specified	Mental health outcomes in people with MH problems	Policy-level and community interventions
[31]	Qualitative (interviews)	South Africa–National	Mental healthcare providers: purposive sampling	Semi-structured interviews	Attitudes toward exercise for mental illness	Advocacy for integrating PA into mental healthcare
[36]	Commentary/Model proposal	South Africa		Not applicable	Advocacy and implementation feasibility	Proposes an SA-based sport and exercise psychiatry model for LMICs
[32]	Intervention development study	South Africa–KwaZulu-Natal	Adolescents, community and school sampling	Intervention Mapping Protocol	Physical activity engagement, cultural appropriateness	“Nyakaza-Move-for-Health”–culturally tailored adolescent PA programme
[29]	Cross-sectional	South Africa–Eastern Cape	Adolescents (*n* ≈ 300); stratified sampling	Body composition measures, PA recall, mental well-being questionnaire	Mental well-being, PA levels, and BMI	Observational–no direct intervention
[27]	Cross-sectional	South Africa–Soweto (urban)	Young women (18–25 years); cohort-based sampling	GHQ-28, accelerometers, sleep logs	Mental health indicators, PA/sedentary behaviour	Observational: highlights associations
[33]	Systematic review & meta-analysis	Global	RCTs in children and adolescents	HRQoL scales (PedsQL, SF-36, etc.)	Health-related quality of life (HRQoL)	Evaluated the impact of PA interventions on HRQoL
[28]	Cross-sectional	South Africa–KwaZulu-Natal	Health professionals in rural district hospital *n* = 63); convenience sampling	IPAQ–short form	Physical activity levels among health workers	Observational–no intervention
[35]	Scoping review	South Africa	Youth-focused studies (15–24 years)	Review of national data & literature	Youth mental health trends, interventions	Recommends integrated, culturally relevant mental health/PA models

Notes: PA = Physical Activity; FGDs = Focus Group Discussions; SES = Socioeconomic Status; BMI = Body Mass Index; MVPA = Moderate-to-Vigorous Physical Activity; HDSS = Health and Demographic Surveillance System; MHH = Mental Health and Wellbeing; LMICs = Low- and Middle-Income Countries; HRQoL = Health-Related Quality of Life; IPAQ = International Physical Activity Questionnaire; PedsQL = Pediatric Quality of Life Inventory.

## Data Availability

No new data were created or analyzed in this study. Data sharing is not applicable to this article.

## Supplementary Materials

The following supporting information can be downloaded at: https://www.mdpi.com/article/10.3390/ijerph23020243/s1, Table S1: PRISMA-ScR Checklist.

## Abbreviations

The following abbreviations are used in this manuscript:

PTSD	Post-Traumatic Stress Disorder
PA	Physical Activity
RISMA-ScR	Preferred Reporting Items for Systematic Reviews and Meta-Analyses extension for Scoping Reviews
OSF	Open Science Framework
